# Enhancing PSMA-uptake with androgen deprivation therapy – a new way to detect prostate cancer metastases?

**DOI:** 10.1590/S1677-5538.IBJU.2018.0305

**Published:** 2019-07-27

**Authors:** Conrad Leitsmann, Paul Thelen, Marianne Schmid, Johannes Meller, Carsten-Oliver Sahlmann, Birgit Meller, Lutz Trojan, Arne Strauss

**Affiliations:** 1 Department of Urology, University Medical Center Goettingen, Goettingen, Germany; 2 Department of Nuclear Medicine, University Medical Center Goettingen, Goettingen, Germany

**Keywords:** Prostatic Neoplasms, Radiotherapy, Magnetic Resonance Imaging

## Abstract

**Purpose:**

^68^Ga-PSMA PET/CT imaging is a promising modality for the staging of recurrent prostate cancer (PCa). Current evidence suggests limited diagnostic value of the ^68^Ga-PSMA PET/CT in PSA-levels ≤0.3ng/mL. Experimental data have demonstrated an increase in PSMA-expression in PCa metastases by androgen deprivation *in vitro.* The aim of the current study was to investigate a possible enhancing effect of PSMA with low-dose androgen deprivation in patients with BCR and low PSA-levels.

**Materials and Methods:**

Five patients with PCa and BCR, following radical prostatectomy, underwent ^68^Ga-PSMA PET/CT. A consecutive ^68^Ga-PSMA PET/CT was performed 6 to 11 days after injection of 80mg of Degarelix (Firmagon^®^). We recorded PSA and testosterone serum-levels and changes of PSMA-uptake in ^68^Ga-PSMA PET/CT images. *Results:* Median PSA prior ^68^Ga-PSMA PET/CT was 0.27ng/mL. All patients had a decrease in testosterone serum levels from median 2.95μg/l to 0.16μg/l following Degarelix injection. We observed an increase in the standardized uptake value (SUV) in PSMA-positive lymphogenous and osseous lesions in two patients following androgen deprivation. In another two patients, no PSMA positive signals were detected in either the first or the second scan.

**Conclusion:**

Our preliminary results of this feasibility assessment indicate a possible enhancing effect of PSMA-imaging induced by low-dose ADT. Despite several limitations and the small number of patients, this could be a new approach to improve staging by ^68^Ga-PSMA PET/CT in PCa patients with BCR after primary therapy. Further prospective studies with larger number of patients are needed to validate our findings.

## INTRODUCTION

Prostate cancer (PCa) is the most common male cancer in Europe with increasing incidence in the past two decades (1). Current curative therapy options include radical prostatectomy (RP) and radiation therapy (RT) (2). However, up to 40% of patients develop biochemical recurrence (BCR) within 10 years after primary therapy (3). Within this context, detection of metastases of PCa is challenging for imaging methods (4). The sensitivity and specificity of conventional imaging such as computed tomography (CT) and magnetic resonance imaging (MRI) are limited (5).

Increasingly discussed salvage treatment of recurrent PCa warrants exact staging (6). Several studies have demonstrated an advantage of Gallium (^68^Ga)-labelled prostate specific membrane antigen (PSMA) positron emission tomography/computed tomography (PET/CT) imaging compared to conventional imaging and functional imaging with ^18^F-choline-based PET/CT for patients with BCR (5,7–12). PSMA, which is a Type-II transmembrane protein, is overexpressed in nearly all PCa cells (13, 14). Studies using ^68^Ga-PSMA PET/CT as the staging modality showed promising results for detecting PCa relapse (13). Current guidelines therefore recommend PSMAPET/CT in patients with PSA recurrence of >1ng/mL after radical prostatectomy (15). In patients with PSA-values between 0.2 and 0.5ng/mL following RP, the reported detection rate of metastases is about 58.0% (9). Other prospective studies showed similar detection rates of 50 to 57% in ^68^Ga-PSMA PET/CT in patients with a PSA-level of ≤0.2ng/mL after RP (7, 16). Nonetheless, these investigations implicate a certain limitation of ^68^Ga-PSMA PET/CT pertaining to the detection of metastases in patients with BCR and low PSA serum-levels after initial treatment.

Androgen deprivation increases PSMA-expression in PCa cells *in vitro* (6, 17). Consequently, this could lead to improved imaging with PSMA-ligand PET/CT (18). The aim of the current study was to investigate a possible enhancing effect of PSMA with low-dose androgen deprivation in patients with BCR and low PSA-levels. We hypothesized that androgen deprivation prior to a ^68^Ga-PSMA PET/CT could increase PSMA expression and therefore the uptake of the ^68^Ga-PSMA-tracer in primarily not evident PCa metastases and to consequently improve the accuracy of staging in BCR patients.

## MATERIALS AND METHODS

### Patients

Five PCa patients with BCR following RP were included in this “feasibility assessment” between February 2016 and September 2016 at our department. BCR was defined as two consecutive PSA-values of ≤0.2ng/mL and rising (15). For each patient, age, initial PSA-value, year of RP, pathological tumour stage (19), Gleason score, time since RP, course of adjuvant RT, PSA-value prior to the first scan and after androgen deprivation, and serum testosterone levels were available. No anti-androgen treatment was recorded for any patient before or at the moment of study inclusion. The study was conducted on the basis of a compassionate use approach.

### ^68^Ga-PSMA PET/CT Imaging

We performed a baseline ^68^Ga-PSMA PET/CT to evaluate the current status of the disease. A consecutive ^68^Ga-PSMA PET/CT was performed after androgen deprivation therapy (ADT) to measure changes in the uptake of PSMA, per standardized uptake value (SUV). We compared the SUV in the baseline PET/CT with the consecutive PET/CT scan following androgen deprivation. The ^68^Ga-PSMA PET/CT was performed as previously described (12, 20). An experienced nuclear medicine physician analysed the images. The SUVmax 1 hour post injection was recorded.

#### Androgen deprivation

All patients received a subcutaneous injection of 80mg Degarelix (Firmagon^®^), a gonadotropin-releasing hormone (GnRH) antagonist that lowers the testosterone serum level below castration level by blocking the GnRH receptor (21). However, there is no recommendation of Degarelix-dosage to increase the PSMA-uptake of PCa metastases. Therefore, we decided to start with 80mg of Degarelix to evaluate any effect mirrored in the PSMA-PET/CT without inducing a curative and thus “vanishing” effect of Degarelix on possible lesions. Two patients with no PSMA uptake in the first scan received Dagarelix as well with the intention to “unmask” possible lessons in the second scan. In our cohort, Degarelix lowered the serum testosterone level under the level of 0.5μg/L within 3 days in all patients (21). We decided to perform the second ^68^Ga-PSMA PET/CT after 6 to 11 days to ensure testosterone levels below castration levels in all patients. The time interval between the injection of ADT and the second ^68^Ga-PSMA-PET/CT was median 9 days. We decided to use this short time frame to avoid a prolonged and potentially “curative” effect of Degarelix that would mask potential metastatic lesions.

### Statistical analysis

Descriptive statistics of variables focused on frequencies. Means and standard deviations, medians and interquartile ranges were reported. Covariates consisted of age, initial PSA-value, year of the RP, pathological tumour stage (19), Gleason score, time since the RP, course of adjuvant RT, PSA-value prior to the first scan and after androgen deprivation, and serum testosterone levels.

All analyses were performed using Statistical Package for the Social Sciences (SPSS, Inc., Chicago, IL, version 23). All parameters were analysed with the Fisher exact test.

## RESULTS

### Study cohort

We numbered patients from No. 1 to 5. Patient characteristics are displayed in Table-1. Median age was 65 years (range 63-70 years), median PSA-value before RP was 10.0ng/mL (range 9.2 -11.54ng/mL). All patients had a high-risk PCa for BCR according to EAU risk groups and two of them had undergone adjuvant RT after RP (15). Median PSA prior the first ^68^Ga-PSMA PET/CT was 0.27ng/mL (range 0.24 -1.76ng/mL). The time interval between RP and the first ^68^Ga-PSMA PET/CT was a median of 37 months (range 23-95 months). The time interval between the first ^68^Ga-PSMA PET/CT and the second was a median of 50 days (range 30-82 days). The time between the injection of Degarelix and the second scan was a median of 9 days (range 6-11 days). All time intervals per patient are shown in Table-2.

**Table 1:** Patients' characteristics (n=5).

	iPSA (ng/mL)	Age (years)	pT	Gleason score	pN1	R1	High risk	Adjuvant radiation
Patient 1	10.5	70	pT3b	4+3	0	1	1	1
Patient 2	9.2	65	pT2c	3+4	0	1	1	0
Patient 3	9.5	63	pT3b	4+3	1	1	1	1
Patient 4	11.5	65	pT2c	3+4	0	0	1	0
Patient 5	9.5	66	pT2c	3+3	0	0	1	0
Median (range)	10.048 (9.2-11.54)	65.8 (63-70)						
Mean (standard deviation)	9.5 (±0.96)	65 (±2.6)						

**Table 2:** Time intervals.

	Time between injection and second scan (days)	Time between RP and the first scan (months)	Operation date
Patient 1	6	37	Jan 13
Patient 2	11	29	Sep 13
Patient 3	11	23	May 14
Patient 4	8	71	Dec 09
Patient 5	9	95	Sep 08
Median (range)	9 (6-11)	51 (23-95)	
Mean (standard deviation)	9 (±2.12)	37 (±30.1)	

### Androgen deprivation

All patients showed a reduction of testosterone serum levels (median 0.16μg/L) after Degarelix injection. The initial PSA values (median of 0. 27ng/mL) prior to androgen deprivation decreased by a median of 0.1ng/mL (range 0.02 -0.94ng/mL, Table-3 and Table-4).

**Table 3:** PSA and testosterone levels before and after androgen deprivation (n=5).

Variables	1 - Scan median (mean)	2 - Scan median (mean)	Difference median (mean)
PSA-Level (ng/mL)	0.27 (0.632)	0.25 (0.32)	0.1 (0.312)
Testosterone (μg/I)	2.95 (3.244)	0.16 (0.174)	2.85 (3.07)
Androgen index	34 (33.6)	2 (2)	32 (31.6)

**Table 4:** PSA-Levels.

	PSA before first scan (ng/mL)	PSA before second scan (ng/mL)	Difference (ng/mL)
Patient 1	0.7	0.26	0.44
Patient 2	0.27	0.25	0.02
Patient 3	1.76	0.82	0.94
Patient 4	0.19	0.13	0.06
Patient 5	0.24	0.14	0.1

### ^68^Ga-PSMA PET/CT Imaging

**Figure 1:**
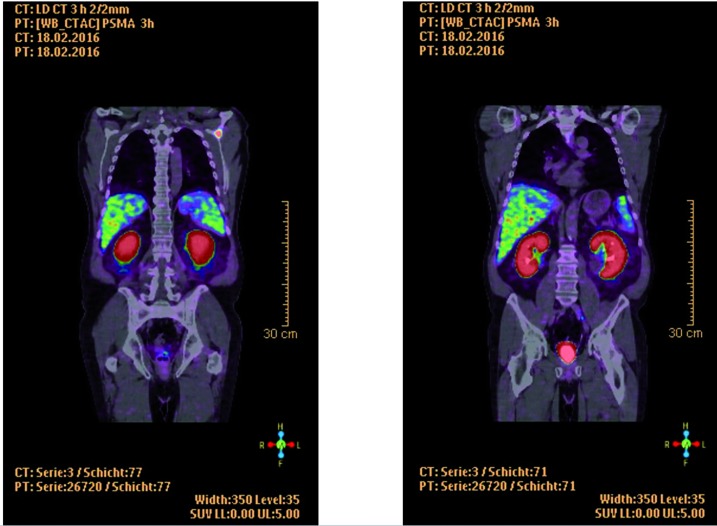
Patient 1, first scan, left: osseous metastasis in the scapula, right: lymph node in the left iliac region.

**Figure 2:**
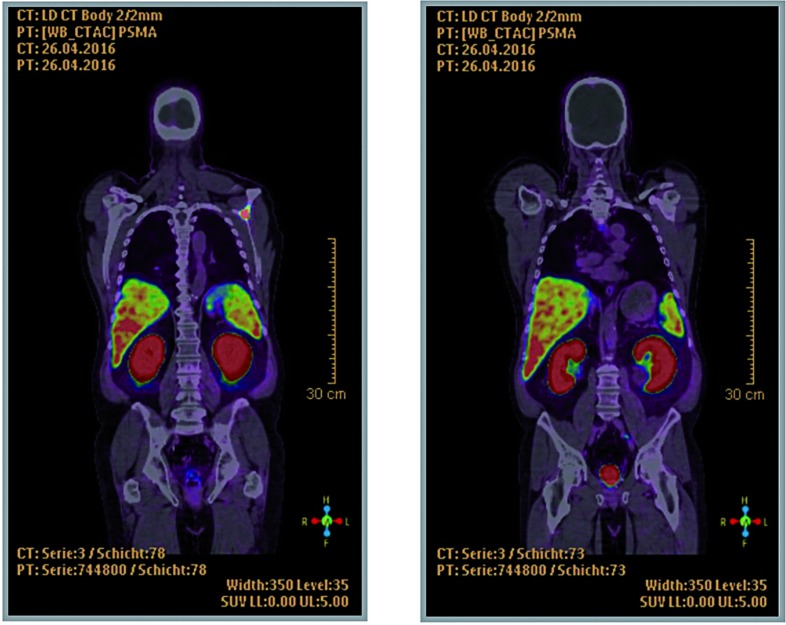
Patient 1, second scan, left: osseous metastasis in the scapula, right: lymph node in the left iliac region.

Three of five patients had a suspect lesion and thus an uncertain tumour manifestation in the first ^68^Ga-PSMA PET/CT. Patients No. 1 and 2 had an increase in PMSA-uptake in the second ^68^Ga-PSMA PET/CT. In the baseline PSMA PET/CT of patient No. 1, we detected one iliac lymphogenous and one osseous lesion in the scapula. SUV of the lymph node in the left iliac region increased from 3.0 in the first scan to 3.8 in the second scan (Figures 1 and 2). The left scapula metastasis measured a SUV of 7.9 and 9.6 in the first and second ^68^Ga-PSMA PET/CT, respectively. Patient No. 2 had an osseous uptake in the right ileum bone with a change in SUV from 1.7 to 1.9 in the first and second ^68^Ga-PSMA PET/CT, respectively.

In patient No. 3, we detected a suspect pulmonary lesion which showed a decrease in SUV from 9.3 to 5.0. A trans-bronchial puncture of this lesion showed no evidence of a PCa metastasis, but the pathology report described “some inflammatory changes of the tissue”. Patients No. 4 and 5 showed no metastasis-suspect lesions in either the first nor the second ^68^Ga-PSMA PET/CT (Table-5).

**Table 5:** Findings PSMA-PET-CT.

	Lesion	1-SUV	2-SUV	Difference
Patient 1	iliac lymph node	3	3.8	+0.8
	osseous metastasis in the scapula	7.9	9.6	+1.7
Patient 2	osseous metastasis of the right ileum	1.7	1.9	+0.2
Patient 3	pulmonary lesion	9.3	5	−4.3
Patient 4	None	-	-	-
Patient 5	None	-	-	-

## DISCUSSION

The aim of our investigation was to assess the feasibility of a possible enhancing effect of low-dose ADT on the PSMA-uptake to improve the detection of PCa metastases in patients with BCR and low PSA-values. In one of our recent studies, androgen deprivation showed a positive effect on *in vitro* PSMA-upregulation. Based on this information, we aimed to increase the PSMA-uptake of metastatic PCa cells in ^68^Ga-PSMA PET/CT imaging, which seems to be limited in low PSA-levels (18). Overall, our preliminary results revealed that, after application of Degarelix, two of five patients showed a SUV increase in a consecutive ^68^Ga-PSMA PET/CT.

The current observation replicates the previously shown *in vitro* upregulation of PSMA-expression following androgen deprivation using Abiraterone acetate (18). A significantly increased uptake of ^68^Ga-PSMA after androgen deprivation was demonstrated in three different cell lines (castration-resistant PCa, revert castration-resistant PCa, and Abiraterone acetate tolerant castration-resistant PCa). Additionally, Wright et al. observed an increase in PSMA expression in primary tissue of the prostate and the post-treatment metastatic specimens following anti-androgen therapy (e.g. orchiectomy, Zoladex^®^, Casodex^®^, Lupron^®^ or Flutamide) (22). Another investigation in cell and animal models suggested a possible response of ^68^Ga-PSMA PET/CT imaging to androgen receptor inhibition (17). Hope et al. recently recapitulated this effect in humans (17). A 51-year old patient received ADT with a single injection of 7.5mg Leuprolide acetate. The patient was imaged on a 3.0 Tesla PET/MRI scanner, once before initiation of ADT and 4 weeks after. A 7-fold higher post-ADT increase in ^68^Ga-PSMA-11 SUV was demonstrated (17). Ceci et al. showed falling PSA-levels in four patients with castration-sensitive disease after ADT in a multivariate subpopulation analysis of 70 patients. All four patients had PSMA positive PET/CT with loco regional lymph nodes or osseous metastases (8). Our results are corroborated by these previous studies that indicate a new approach to increasing the sensitivity of ^68^Ga-PSMA PET/CT for staging by administering ADT in patients with recurrent PCa and low PSA-levels.

We included patients with a median PSA serum-level of 0.27ng/mL. For a comparable group of patients, Eiber et al. and others showed a detection rate for metastases between 50.0-58.0% (7, 9, 16). However, for patients with BCR at low PSA serum-levels, accurate staging is required in order to discuss therapeutic salvage options such as surgery or RT. Current guidelines suggests a salvage RT for patients with BCR after RP with a PSA serum-level <0.5ng/mL (23). There is no Level 1 evidence for a benefit and therefore no recommendation to perform a salvage lymphadenectomy (23). Some studies have indeed shown promising data for salvage lymphadenectomy after a ^68^Ga-PSMA PET/CT (2, 20, 24–27). Rauscher et al. showed a possible opportunity using ^68^Ga-PSMA PET/CT for choosing a curative treatment like a salvage RT or a salvage lymphadenectomy or even a PSMA-radio-guided surgery (23, 28). As a result, a possible enhancing effect of PSMA could improve imaging for staging of patients with recurrent PCa after primary therapy such as RP and consequently support the opportunity for choosing an additional (curative) treatment.

Patients No. 1 and No. 3 underwent adjuvant RT following RP. Patient No. 1 showed an increase in PSMA after androgen deprivation but Patient No. 3 did not. So, it seems that the fact of a prior adjuvant RT had no influence on the uptake of PSMA in the second ^68^Ga-PSMA PET/CT. However, RT could possibly influence a PSMA boosting with androgen deprivation. Further investigations are needed to evaluate the influence of adjuvant RT.

Patient No. 3 had a pulmonary lesion that stayed unclear. The second ^68^Ga-PSMA PET/CT showed a decrease in the uptake. Therefore, it is unclear if this was a metastasis of a PCa or another tissue which had a PSMA-expression, as described in other publications. In fact, PSMA positivity may also appear in inflammation or after trauma (29).

Hope et al. suggested two causal processes for the increased PSMA with ADT (17): first, the increase in PSMA expression is due to androgen receptor inhibition showed by a recent study (30). Second, there is a decrease of tumour mass by a therapeutically induced cell death with ADT (31).

The question of the optimal androgen deprivation and the timeframe between the application and the scan stays unclear after our first study. Abiraterone acetate could be a good alternative drug for further investigations. *In vivo,* we could show an enhanced PSMA-expression in PCa cells with Abiraterone acetate after 48 hours. A shorter timeframe between the application of anti-androgen therapy and ^68^Ga-PSMA PET/CT should be discussed (18). Hope et al. and Ceci et al. showed an increase in PSMA in PET/CT after 4 weeks or longer of androgen deprivation (8, 17, 31). ADT has never been used for this purpose before in humans and therefore no explicit recommendations exist.

Our study is certainly not devoid of limitations. First, the number of patients is limited which makes a strong conclusion difficult, considering no statistically significant results could be obtained. Further, only 3 of our patients had low PSA levels between 0.19 and 0.27ng/mL, whereas the 2 other patients had higher PSA serum levels. Although recommended by current guidelines (15), in Germany PSMA-PET/CT as imaging modality in such a setting is not covered by public health insurances and therefore not frequently performed. In clinical practice PSMA-PET/CT is therefore performed as self-payment or justified as an individual diagnostic pathway. In addition, there is no clear definition of “low” PSA-levels in the setting of BCR. For further investigations we will be more stringent and plan to include only patients with PSA-levels <0.5ng/mL. Second, we only observed an effect in two of five patients after the application of Degarelix. One patient, however, showed a mixed response and two patients showed no effect. This investigation is the first who reports on the effect of low dose Degarelix on PSMA uptake. However, data is preliminary and the study was performed as an individual diagnostic pathway per patient. Further investigations are needed to include more patients to evaluate its validity. Third, we observed a SUV of metastases from the first to the second scan, but we could not detect new, primarily “hidden” metastases. Further studies are needed to establish the appropriate dosage of ADT that has the effect of detecting PCa lesions.

Further investigations with a change of protocol, using different ADT such as Abiraterone or another timeframe between the application and the ^68^Ga-PSMA PET/CT need to be performed. Furthermore, specific analysis will need to focus on patients following RT.

## CONCLUSIONS

Our preliminary results of this feasibility assessment indicate a possible enhancing effect of PSMA-imaging induced by low-dose ADT. Despite several limitations and the small number of patients, this could be a new approach to improve staging by ^68^Ga-PSMA PET/CT in PCa patients with BCR after primary therapy. Consequently, better staging translates into improved therapy options for these patients. Further prospective studies with larger number of patients are needed to validate our findings.

### Compliance with Ethical Standards

Research involving human participants and/or animals:

All procedures performed in studies involving human participants were in accordance with the ethical standards of the institutional and/or national research committee and with the 1964 Helsinki declaration and its later amendments or comparable ethical standards.

For this type of study formal consent is not required.

This article does not contain any studies with animals performed by any of the authors

Informed consent was obtained from all individual participants included in the study.

## References

[1] Bray F, Lortet-Tieulent J, Ferlay J, Forman D, Auvinen A (2010). Prostate cancer incidence and mortality trends in 37 European countries: an overview. Eur J Cancer..

[2] Mottet N, Bellmunt J, Bolla M, Briers E, Cumberbatch MG, De Santis M (2017). EAU-ESTRO-SIOG Guidelines on Prostate Cancer. Part 1: Screening, Diagnosis, and Local Treatment with Curative Intent. Eur Urol..

[3] Roehl KA, Han M, Ramos CG, Antenor JA, Catalona WJ (2004). Cancer progression and survival rates following anatomical radical retropubic prostatectomy in 3,478 consecutive patients: long-term results. J Urol..

[4] Kosuri S, Akhtar NH, Smith M, Osborne JR, Tagawa ST (2012). Review of salvage therapy for biochemically recurrent prostate cancer: the role of imaging and rationale for systemic salvage targeted anti-prostate-specific membrane antigen radioimmunotherapy. Adv Urol..

[5] Afshar-Oromieh A, Avtzi E, Giesel FL, Holland-Letz T, Linhart HG, Eder M (2015). The diagnostic value of PET/CT imaging with the (68)Ga-labelled PSMA ligand HBED-CC in the diagnosis of recurrent prostate cancer. Eur J Nucl Med Mol Imaging..

[6] Anderström C, Johansson SL, von Schultz L (1983). Primary adenocarcinoma of the urinary bladder. A clinicopathologic and prognostic study. Cancer..

[7] Afshar-Oromieh A, Zechmann CM, Malcher A, Eder M, Eisenhut M, Linhart HG (2014). Comparison of PET imaging with a (68)Ga-labelled PSMA ligand and (18)F-choline-based PET/CT for the diagnosis of recurrent prostate cancer. Eur J Nucl Med Mol Imaging..

[8] Ceci F, Uprimny C, Nilica B, Geraldo L, Kendler D, Kroiss A (2015). (68)Ga-PSMA PET/CT for restaging recurrent prostate cancer: which factors are associated with PET/CT detection rate?. Eur J Nucl Med Mol Imaging..

[9] Eiber M, Maurer T, Souvatzoglou M, Beer AJ, Ruffani A, Haller B (2015). Evaluation of Hybrid ^68^Ga-PSMA Ligand PET/CT in 248 Patients with Biochemical Recurrence After Radical Prostatectomy. J Nucl Med..

[10] Flanigan RC, McKay TC, Olson M, Shankey TV, Pyle J, Waters WB (1996). Limited efficacy of preoperative computed tomographic scanning for the evaluation of lymph node metastasis in patients before radical prostatectomy. Urology..

[11] Morigi JJ, Stricker PD, van Leeuwen PJ, Tang R, Ho B, Nguyen Q (2015). Prospective Comparison of 18F-Fluoromethylcholine Versus 68Ga-PSMA PET/CT in Prostate Cancer Patients Who Have Rising PSA After Curative Treatment and Are Being Considered for Targeted Therapy. J Nucl Med..

[12] Hijazi S, Meller B, Leitsmann C, Strauss A, Ritter C, Lotz J (2016). See the unseen: Mesorectal lymph node metastases in prostate cancer. Prostate..

[13] Eder M, Schäfer M, Bauder-Wüst U, Hull WE, Wängler C, Mier W (2012). 68Ga-complex lipophilicity and the targeting property of a urea-based PSMA inhibitor for PET imaging. Bioconjug Chem..

[14] Perera M, Papa N, Christidis D, Wetherell D, Hofman MS, Murphy DG (2016). Sensitivity, Specificity, and Predictors of Positive (68)Ga-Prostate-specific Membrane Antigen Positron Emission Tomography in Advanced Prostate Cancer: A Systematic Review and Meta-analysis. Eur Urol..

[15] Mottet N, Bellmunt J, Bolla M, Briers E, Cumberbatch MG, De Santis M (2017). EAU-ESTRO-SIOG Guidelines on Prostate Cancer. Part 1: Screening, Diagnosis, and Local Treatment with Curative Intent. Eur Urol..

[16] van Leeuwen PJ, Stricker P, Hruby G, Kneebone A, Ting F, Thompson B (2016). (68) Ga-PSMA has a high detection rate of prostate cancer recurrence outside the prostatic fossa in patients being considered for salvage radiation treatment. BJU Int..

[17] Hope TA, Truillet C, Ehman EC, Afshar-Oromieh A, Aggarwal R, Ryan CJ (2017). 68Ga-PSMA-11 PET Imaging of Response to Androgen Receptor Inhibition: First Human Experience. J Nucl Med..

[18] Meller B, Bremmer F, Sahlmann CO, Hijazi S, Bouter C, Trojan L (2015). Alterations in androgen deprivation enhanced prostate-specific membrane antigen (PSMA) expression in prostate cancer cells as a target for diagnostics and therapy. EJNMMI Res..

[19] Brierley JD, Gospodarowicz MK, Wittekind C (2016). TNM classification of malignant tumours.

[20] Hijazi S, Meller B, Leitsmann C, Strauss A, Meller J, Ritter CO (2015). Pelvic lymph node dissection for nodal oligometastatic prostate cancer detected by -PSMA-positron emission tomography/computerized tomography. Prostate..

[21] Klotz L, Boccon-Gibod L, Shore ND, Andreou C, Persson BE, Cantor P (2008). The efficacy and safety of degarelix: a 12-month, comparative, randomized, open-label, parallel-group phase III study in patients with prostate cancer. BJU Int..

[22] Wright GL, Grob BM, Haley C, Grossman K, Newhall K, Petrylak D (1996). Upregulation of prostate-specific membrane antigen after androgen-deprivation therapy. Urology..

[23] Cornford P, Bellmunt J, Bolla M, Briers E, De Santis M, Gross T (2017). EAU-ESTRO-SIOG Guidelines on Prostate Cancer. Part II: Treatment of Relapsing, Metastatic, and Castration-Resistant Prostate Cancer. Eur Urol..

[24] Heidenreich A, Moul JW, Shariat S, Karnes RJ (2016). Role of salvage lymph node dissection in prostate cancer. Curr Opin Urol..

[25] Pfister D, Porres D, Heidenreich A, Heidegger I, Knuechel R, Steib F (2016). Detection of recurrent prostate cancer lesions before salvage lymphadenectomy is more accurate with (68) Ga-PSMA-HBED-CC than with (18)F-Fluoroethylcholine PET/CT. Eur J Nucl Med Mol Imaging..

[26] Rauscher I, Maurer T, Beer AJ, Graner FP, Haller B, Weirich G (2016). Value of 68Ga-PSMA HBED-CC PET for the Assessment of Lymph Node Metastases in Prostate Cancer Patients with Biochemical Recurrence: Comparison with Histopathology After Salvage Lymphadenectomy. J Nucl Med..

[27] Schiavina R, Ceci F, Romagnoli D, Uprimny C, Brunocilla E, Borghesi M (2015). (68)Ga-PSMA-PET/CT-Guided Salvage Retroperitoneal Lymph Node Dissection for Disease Relapse After Radical Prostatectomy for Prostate Cancer. Clin Genitourin Cancer..

[28] Rauscher I, Düwel C, Wirtz M, Schottelius M, Wester HJ, Schwamborn K (2017). Value of (111) In-prostate-specific membrane antigen (PSMA)-radioguided surgery for salvage lymphadenectomy in recurrent prostate cancer: correlation with histopathology and clinical follow-up. BJU Int..

[29] Kinoshita Y, Kuratsukuri K, Landas S, Imaida K, Rovito PM, Wang CY (2006). Expression of prostate-specific membrane antigen in normal and malignant human tissues. World J Surg..

[30] Vallabhajosula S, Jhanwar Y, Tagawa S (2016). 99mTc-MIP-1404 Planar and SPECT scan: Imaging biomarker of androgen receptor (AR) signaling and prostate specific membrane antigen (PSMA) expression. Journal of Nuclear Medicine..

[31] Schlenkhoff CD, Gaertner F, Essler M, Hauser S, Ahmadzadehfar H (2016). 68Ga-Labeled Anti-Prostate-Specific Membrane Antigen Peptide as Marker for Androgen Deprivation Therapy Response in Prostate Cancer. Clin Nucl Med..

